# Der Gasteiner Heilstollen und eine mögliche Ansteckungsgefahr im Therapiebereich mit Viren

**DOI:** 10.1007/s12688-020-00350-6

**Published:** 2020-06-23

**Authors:** M. Offenbächer, B. Hölzl, M. Gaisberger, H. Untner, R. Würzner

**Affiliations:** 1 Gasteiner Heilstollen, Heilstollenstr. 19, Bad Gastein-Böckstein, Österreich; 2 Landesklinik St. Veit, St. Veit, Österreich; 3 Institut für Physiologie und Pathophysiologie und Forschungsinstitut Gastein, Paracelsius Medizinische Universität, Salzburg, Österreich; 4 Institut für Hygiene und Medizinische Mikrobiologie, Medizinische Universität, Innsbruck, Österreich

**Keywords:** COVID-19, Klimatische Faktoren, Virusübertragung, Milde Radonhyperthermie, Immunsystem, COVID-19, Climatic factors, Virus transmission, Low dose radon hyperthermia, Immune system

## Abstract

An SARS-CoV‑2 haben sich in den letzten Monaten weltweit Millionen von Menschen infiziert, Hunderttausende sind an den Folgen einer Infektion gestorben. Das Ende der Pandemie ist nicht absehbar und viele Menschen haben Ängste, sich in unterschiedlichen Settings zu infizieren. Der Gasteiner Heilstollen (GHST) ist eine weltweit einmalige ambulante Einrichtung mit den Wirkfaktoren Wärme, hohe Luftfeuchtigkeit und milde Radonstrahlung. In dieser werden jährlich ca. 12.000 Patienten u. a. mit entzündlich-rheumatischen, degenerativen Erkrankungen und chronischen Schmerzen behandelt. Wir haben deshalb die Literatur gesichtet und hinsichtlich einer möglicherweise erhöhten Ansteckungsgefährdung für Patienten während einer Therapie bzw. Kur im GHST analysiert. Aus unserer Sicht sind zum einen die klimatischen und physikalischen Verhältnisse im GHST insgesamt als *virenfeindlich* anzusehen, zum anderen führen die milde Radonhyperthermie über komplexe physiologische Prozesse sowie die geografische Lage des GHST zu positiven gesundheitlichen Effekten. Daher erscheint uns eine Ansteckungswahrscheinlichkeit für Viren im GHST in keinem Fall erhöht, sondern sogar deutlich geringer als in anderen Settings.

## Abstract (en)

The SARS-CoV‑2 has infected millions of humans worldwide in the past few months and hundreds of thousands have died as a result of an infection. The end of the pandemic is not in sight and many people are anxious of becoming infected in different settings. The Gastein Healing Gallery (GHG) is a unique outpatient facility combining heat, high humidity and mild radon radiation. Every year approximately 12,000 patients with inflammatory rheumatic, degenerative diseases and chronic pain are treated. We have therefore reviewed and analyzed the literature with respect to a possible increased risk of infection for patients during treatment in the GHG. On the one hand the climatic and physical conditions in the GHG can be viewed as hostile to viruses and on the other hand the mild radon hyperthermia and the geographic location of the GHG lead to positive effects on the patient’s health via complex physiological processes. We therefore consider the likelihood of infection with viruses in the GHG in no way increased, in contrast, it is probably considerably lower compared to other settings.

SARS-CoV‑2 gehört zu der Gruppe der Coronaviren und ist für den menschlichen Organismus noch unbekannt. Deshalb weiß man noch relativ wenig über sein Verhalten, die genauen Übertragungs- und Infektionsmechanismen. Die wissenschaftlichen Erkenntnisse zu den verschiedenen Aspekten werden jedoch von Tag zu Tag mehr und vieles ist auch von den SARS-CoV‑2 verwandten Viren (SARS, MERS) oder Influenza, die schon länger Menschen infizieren, bekannt.

Das klinische Spektrum einer Infektion mit SARS-CoV‑2 reicht von einem asymptomatischen Verlauf, über milde Symptome wie z. B. trockenem Husten, Halsschmerzen, Fieber und leichter Atemnot bis zu schweren Ausprägungen mit einer viralen Pneumonie, akutem respiratorischem Distress-Syndrom (ARDS) und nachfolgendem Organversagen [1]. Ein ARDS erfordert eine Sauerstoffgabe sowie eine Beatmung des Patienten, und trotz dieser Maßnahmen ist die Mortalität hoch. Die große Anzahl an Patienten in kurzer Zeit, die eine Beatmung benötigten, brachten während der Pandemie in einer Reihe von Ländern und Regionen die Gesundheitssysteme an den Rand des Zusammenbruchs [2–4].

Das Virus hat eine Größe von 60–160 nm. Die Übertragung zwischen infizierten und gesunden Mensch findet am häufigsten über Tröpfchen, die beim Niesen, Husten, Singen, Sprechen und Ausatmen entstehen, statt. Diese Tröpfchen sind sehr klein und bleiben, je nach Größe und je nach den Umweltbedingungen, unterschiedlich lange in der Luft [5]. Neben den bekannten hygienischen Maßnahmen haben auch Umweltbedingungen nachweislich einen Einfluss auf die Inaktivierung von Viren. Auf Grund der momentanen Situation mit einer potenziellen Infektionsgefahr in unterschiedlichen Settings mit SARS-CoV‑2 entwickeln viele Menschen Ängste [6], die wiederum negative Effekte auf das Immunsystem haben und damit ein Infektionsrisiko erhöhen [7] können. Daher war unser Ziel, die Literatur zu sichten und hinsichtlich einer möglicherweise erhöhten Ansteckungsgefährdung für Patienten während einer Therapie bzw. Kur im Gasteiner Heilstollen (GHST) in Bad Gastein zu analysieren.

## Einfluss von Temperatur und Luftfeuchtigkeit auf Viren

Das Klima im GHST ist feucht und warm, von 37° bei 75 % Luftfeuchtigkeit auf Station 1 bis zu 41° bei fast 100 % Luftfeuchtigkeit auf Station 4. Klimatische Verhältnisse haben einen negativen Einfluss auf die Übertragungs- und Infektionsrate von Viren. Dies wurde in wissenschaftlichen Studien untersucht und nachgewiesen. So haben kürzlich publizierte Studien gezeigt, dass die Übertragungsrate stark von Temperatur und Luftfeuchtigkeit abhängt:

Wang und Mitarbeiter [8] berichteten, dass eine Erhöhung um 1 Grad Lufttemperatur und 1 % mehr Luftfeuchtigkeit die effektive Reproduktionsrate R um 0,0225 bzw. 0,0158 von SARS-CoV‑2 reduziert. Laut den Autoren sind ihre Befunde konsistent mit der Übertragungsrate von Influenza und erklärt sich folgendermaßen: Viele Viren sind stabiler bei kalten Temperaturen und die Tröpfchen, die das Virus enthalten, bleiben bei trockener Kälte länger in der Luft. Des Weiteren führt kalte, trockene Luft zu einer Schwächung des Immunsystems und macht dadurch den Organismus anfälliger für eine Infektion.

Ängste haben negative Effekte auf das Immunsystem und können ein Infektionsrisiko erhöhen

Liu und Kollegen [9] untersuchten in 17 chinesischen Städten, die mehr als 50 bestätigte SARS-CoV‑2 Fälle dokumentierten, den Einfluss von meteorologischen Faktoren. Die Autoren schlossen aus ihren Ergebnissen, dass lokale meteorologische Konditionen mit einer niedrigen Temperatur, einer geringen Temperaturschwankung am Tage sowie einer niedrigen Luftfeuchtigkeit die Übertragung vermutlich verstärkt.

Auch das Team um Araujo [10] geht davon aus, dass das Auftreten von SARS-CoV 2 einem saisonalen Muster folgt: vermehrtes Auftreten bei kaltem und trockenen Wetter, Verlangsamung der Ausbreitung bei heißem und feuchten Wetter. Der Mechanismus, der diesen klimatischen Einfluss zu Grunde liegt, hängt wahrscheinlich mit der Überlebensfähigkeit des Virus unter diesen klimatischen Bedingungen zusammen, bevor dieses einen Menschen infizieren kann. Die Autoren zitieren eine Studie von Chan und Mitarbeitern [11], die zeigte, dass getrocknetes Virenmaterial (in diesem Fall SARS-CoV-1) auf glatter Oberflächen bei einer Temperatur zwischen 11° und 25° und relativer Luftfeuchtigkeit von 40–50 % fünf Tage lang nachzuweisen ist, aber bei steigender Temperatur und Luftfeuchtigkeit drastisch an Überlebensfähigkeit verliert. Die lipidhaltige Schutzhülle des Coronavirus bricht leicht bei höheren Temperaturen auf und eine höhere Luftfeuchtigkeit beeinflusst die Übertragungsrate respiratorischer Viren von Mensch zu Mensch negativ (z. B. schnelleres Zu-Boden-Sinken der *größeren* Tröpfchen).

Eine Studie mit Grippeviren [12] zeigte, dass die relative Luftfeuchtigkeit ein wichtiger Faktor für die Übertragung der Viren ist. Sie hatte zwar nur einen geringen Einfluss auf die Zahl der Viren, die Infektiosität der Viren war jedoch bei einer niedrigen Luftfeuchtigkeit deutlich höher. Wurden die Viren in der Studie in einem Raum mit einer relativen Feuchtigkeit von ≤23 % freigesetzt, behielten nach 60 min noch 70,6–77,3 % ihre Infektionsfähigkeit. Lag die relative Luftfeuchte bei ≥43 %, konnten nur noch 14,6–22,2 % der Viren Zellen infizieren. Der größte Rückgang der Infektionsfähigkeit trat dabei bereits innerhalb der ersten 15 min auf, unabhängig von der Tröpfchengröße. Nach einer Stunde waren bei einer relativen Luftfeuchtigkeit von 45 % keine infektiösen Viren mehr nachweisbar. Die Forscher schlossen daraus, dass eine hohe Luftfeuchte die Viren direkt nach dem Freisetzen inaktiviert und dass eine hohe Luftfeuchtigkeit die Ansteckungsrate bei der Grippe deutlich senkt. Auch eine endemische Ausbreitung über Tröpfcheninfektion ist aus Sicht der Autoren so deutlich erschwert [9].

Die relative Luftfeuchtigkeit ist ein wichtiger Faktor für die Übertragung der Viren

In einem Tierversuch untersuchte die Forschungsgruppe um Lowen [13] die Übertragungsrate von Influenza von einem infizierten Tier auf ein gesundes Tier in Abhängigkeit von der Luftfeuchtigkeit (20–80 %). Die Temperatur lag im Experiment konstant bei 20°. In ihrer Studie war die Übertragungsrate zwischen 75 % und 100 % bei einer Luftfeuchtigkeit von 20 %, 35 %, und 65 %, aber nur 25 % bei 50 % und 0 % bei 80 % Luftfeuchtigkeit. Die Autoren schlossen aus ihren Ergebnissen, dass, um die Übertragungsrate von Influenza zu minimieren, die Temperatur über 20° betragen und die Luftfeuchtigkeit bei 50 % bzw. 80 % liegen sollte.

Zusammenfassend lässt sich aus unserer Sicht aus den vorliegenden Erkenntnissen schließen, dass die klimatischen Verhältnisse in den Therapiebereichen des GHST ausgesprochen *virenfeindlich* sind. Daher erscheint uns eine Ansteckungswahrscheinlichkeit für Viren im GHST in keinem Fall erhöht, sondern sogar deutlich geringer als in der Umgebung außerhalb.

Hinzu kommt noch, dass durch die feuchte Wärme im GHST die Schleimhäute, die Haupteintrittspforte für respiratorische Viren, in Nasen-Rachen-Raum und der Lunge besser durchblutet und befeuchtet werden, was zu einer verstärkten lokalen Abwehrkraft gegenüber diesen Viren führt. Eccles [14] beschreibt in seinem Review, dass eine Auskühlung der Schleimhäute im Nasenrachenraum im Winter zu einer deutlichen lokalen Abwehrschwäche durch Beeinträchtigung der mukoziliären Reinigung und Inhibition der Leukozytenphagozytose führt und dadurch u. a. die Saisonalität von Influenza erklärt.

## Aerobiologie – die Übertragung von Viren und Partikeln durch die Luft

Wie bereits geschildert, ist der Hauptübertragungsweg von SARS-CoV‑2 über Tröpfchen von infizierten Personen [5], möglicherweise wird das Virus auch über Aerosole übertragen [15]. In seinem Übersichtsartikel setzt sich Tellier [16] mit der Übertragung durch Tröpfchen, die Influenzaviren enthalten, auseinander. Prinzipiell bleiben kleine Tröpfchen nur eine bestimmte Zeit in der Luft, weil diese auf Grund ihrer Größe nur langsam zu Boden sinken. Zum Beispiel brauchen Partikel mit 20 µm vier Minuten, um aus drei Meter Höhe zu Boden zu sinken, kleinere Partikel bleiben länger in der Luft, so dauert dies bei 5  µm großen ca. 70 min. Man muss aber auch noch beachten, dass nach dem Ausnießen oder Husten von Tröpfchen unterschiedlicher Größe diese innerhalb von Sekundenbruchteilen Wasser verlieren und somit die Hälfte ihrer ursprünglichen Größe verlieren, d. h. potenziell länger in der Luft schweben. Auch Luftverwirbelungen und klimatische Verhältnisse beeinflussen die Fallgeschwindigkeit.

Niesen produziert ca. 40.000 Partikel und Husten ca. 710 Partikel pro Ereignis

Unterschiedliche menschliche Aktivitäten produzieren mehr oder weniger Tröpfchen. Dies haben Fernstrom und Goldblatt [17] in seinem Übersichtsartikel dargestellt: Niesen produziert ca. 40.000 Partikel und Husten ca. 710 Partikel pro Ereignis, und beim Sprechen werden ca. 36 Partikel pro 100 Worte ausgestoßen. Bei Sprechen steigt die Anzahl der emittierten Tröpfchen mit der Lautstärke [18] und beim lauten Sprechen bzw. Singen wird somit das Risiko einer Infektion, wenn Menschen zusammenstehen, erhöht. Dies sagt jedoch noch nichts über die Infektiosität der Tröpfchen aus, wenn ein Infizierter diese ausstößt. Diesbezüglich spielen noch andere Faktoren eine Rolle, wie z. B. die Menge der Viren in einem Tröpfchen.

Bei Influenza hängt die Schwere der Erkrankung auch von der Virendosis ab, dies konnte auch bei SARS-CoV‑1 nachgewiesen werden [19]. Im GHST, beginnend ab der Bademantelstation, steigt die Luftfeuchtigkeit bis auf fast 100 % an, und es ist zu vermuten, dass durch Niesen oder Husten im Therapiebereich emittierte Partikel rasch auf Wasserdampf in der Luft treffen, dadurch vermutlich an Größe gewinnen und daher aus physikalischen Gründen schneller zu Boden fallen.

Außerdem gilt im GHST seit Jahrzehnten ein Ruhegebot (d. h. Nicht-Sprechen) ab der Einfahrt in den Heilstollen und auch im Therapiebereich. Dies wird von den Patienten in der Regel mehrheitlich eingehalten.

## Einfluss der Radonkonzentration und eine Erhöhung der Körpertemperatur im GHST auf die Virusübertragung und das Immunsystem

Durch das Radon besteht im Therapiebereich eine Alphastrahlenbelastung von 44 kBq/m^3^. Die Alphastrahlung durch das Radon im GHST führt zu einer Luftionisation. Die dadurch entstehenden negativen Ionen haben möglichweise einen inaktivierenden Effekt auf Viren, aber auch einen positiven gesundheitlichen Effekt (siehe Übersichtsartikel von Jiang et al. [20]). Darüber hinaus löst die schwach radioaktive Alphastrahlung Prozesse im Organismus aus, die vermutlich eine Immunantwort auf ein Virus unterstützen.

Calabrese und Mitarbeiter publizierten 2019 [21], dass LDR („low dose radiation“) eine hoch integrierte, komplexe und systemische Antwort des Organismus auslöst. In einem Übersichtsartikel zur therapeutischen LDR bei Pneumonien beschrieben sie schon 2013 [22] den Wirkmechanismus. So kommt es nach einer LDR zu einer Reihe von biochemischen und molekularen Ereignissen, die je nach Strahlendosis proinflammatorische bzw. antiinflammatorische Eigenschaften aufweisen. Bei Letzterer kommt es u. a. zu einem Abfall von NO/iNOS, einer Reduktion von ROS, Induktion der Apoptose, Unterdrückung von TGF‑α, sowie einer Verstärkung von T‑regulatorischen Zellen.

Interessanterweise fanden sich in der Studie von Blanco-Melo und Kollegen [23] Hinweise, dass die Immunreaktion des Körpers auf SARS-CoV‑2 nicht balanciert ist und es zu einer proinflammatorischen Antwort mit einer Erhöhung von einer Reihe von Chemokinen und Interleukinen kommt. Die Autoren schlussfolgern, dass bei COVID-19-Patienten möglicherweise Medikamente mit immunmodulierenden Eigenschaften wirksam sein könnten.

Eine klinische positive Wirkung, u. a. im Sinne von weniger entzündlichen Prozessen bzw. der Möglichkeit, Medikamente zu reduzieren berichten uns Patienten mit entzündlich-rheumatischen Erkrankungen regelmäßig nach Heilstolleneinfahrten, was per se auch eine potenzielle Risikoreduktion für eine COVID-19-Infektion bzw. für einen schweren Verlauf dieser darstellen könnte.

Im Gasteiner Heilstollen kommt es in den Therapiebereichen ab der Station Ia zu einer Erhöhung der Kernkörpertemperatur bis zu 39°, was einem künstlichen Fieber entspricht. Letzteres wird vom Körper im Falle einer viralen oder bakteriellen Infektion physiologisch erzeugt, um die Effektivität des Immunsystems zur Bekämpfung der Infektion zu erhöhen. Die Mechanismen, die durch die erhöhte Körpertemperatur ausgelöst werden, sind bisher noch wenig bekannt. Es mehren sich jedoch die Hinweise, dass fieberartige Temperaturen zwischen 38° und 40° eine Rolle spielen, um die Migration von Lymphozyten in Lymphorgane oder Entzündungsgewebe zu erleichtern.

In den Therapiebereichen ab Station Ia wird die Kernkörpertemperatur auf bis zu 39° erhöht

Fieberartiger thermischer Stress verstärkt die endotheliale Expression von ICAM‑1 und Chemokinen (CCL21), was wiederum die Adhäsion der Lymphozyten an sowie den Durchtritt dieser durch die Gefäße („high endothelial venules“ [HEV]) erleichtert [24]. Bei fieberartigen Temperaturen wird auch die L‑Selektin-abhängige Adhäsion der Lymphozyten an die Gefäße stimuliert [25]. Zusammenfassend führen diese physiologischen Mechanismen u. a. dazu, dass bei fieberartigen Körpertemperaturen, wie diese im GHST bei Patienten auftreten, die Migration von Immunzellen zu Entzündungs- bzw. Infektionsherden erleichtert wird und dadurch die Erreger schneller attackiert werden. Weitere positive Effekte der Hyperthermie auf das Immunsystem sind in einem kurzen Übersichtsartikel von Baronzio et al. [26] beschrieben. Obwohl die dargelegten Effekte in der Anflutungsphase sicher COVID-19-inhibierend erscheinen, sind bei einem fortgeschrittenen Stadium der Erkrankung auch Krankheitsverschlechterungen denkbar, weshalb vor Einfahrt in den GHST die Körpertemperatur gemessen wird. Dies ist auch schon zur Verhinderung einer Infektionsübertragung sinnvoll.

## Umweltfaktoren und das Immunsystem

Der Gasteiner Heilstollen liegt auf 1270 m Meereshöhe. Es ist evident, dass die Luftbelastung durch Schadstoffe in der Höhe abnimmt, vor allem wenn keine luftverschmutzende Industrie in der näheren Umgebung zu finden ist, wie z. B. im Gasteiner Tal oder in anderen Kurorten in Österreich. Ebenso nimmt die Allergenbelastung mit zunehmender Höhe ab. Beides, Allergene und Schadstoffe, belasten das Immunsystem und können langfristig zu Erkrankungen führen bzw. die Stressanfälligkeit des Organismus erhöhen. In ihrem Übersichtsartikel beschreiben Glencross und Kollegen [27] die Effekte der Luftverschmutzung auf das Immunsystem. Die Partikel (u. a. Stickstoffdioxid) können direkt zu einer zellulären Signaltransduktion führen: es wurde gezeigt, dass sowohl in Zellkulturen als auch im Tiermodell diese Luftverschmutzungspartikel Effekte auf jede untersuchte Immunzelle hatten. Zusätzlich zu den allgemeinen proinflammatorischen Effekten, die in den Studien nachgewiesen wurden, weisen einige Untersuchungen darauf hin, dass die Partikel die Th2-Immunantwort verstärken und die antimikrobielle Immunreaktion abschwächen. Bemerkenswerterweise wird eine erhöhte chronische Belastung mit Stickoxiden (z. B. in Norditalien bzw. der Region Madrid) mit einer erhöhten Sterblichkeitsrate, zum Teil durch eine überschießende Immunreaktion (*Zytokinsturm*; [28]) ausgelöst, bei einer COVID-19-Infektion in Zusammenhang gebracht [29], da das NO_2_ vermutlich eine (subklinische) Entzündung der Lunge verursacht und somit die Lunge u. a. anfälliger macht für Infekte oder Asthma.

Eine Studie von Arias-Reyes [30] weist darauf hin, dass die Pathogenität von SARS-CoV‑2 in großen Höhen möglicherweise durch physiologische Anpassungsvorgänge des Organismus an die Höhe stark vermindert ist. Auch wenn der physiologische Mechanismus noch unklar ist, ist bekannt, dass eine positive Assoziation zwischen der Infektionsrate mit SARS-CoV‑1 und Angiotension-Convertin Enzym 2 (ACE2) in pulmonalen Epithelzellen besteht. Sowohl SARS-CoV‑1 als auch SARS-CoV‑2 binden sich an ACE2. ACE2 ist ein transmembranöses Enzym in Zellen des Atemtraktes, der Lunge, des Herzens, Arterien, Venen, Niere und des Darmtraktes. Unter chronischer Hypoxie, wie in z. B. in großen Höhen, adaptiert sich der Organismus und ACE1 wird vermehrt gebildet, aber ACE2 dafür weniger in den Zellen der Lunge exprimiert. Das heißt, SARS-CoV‑2 könnte somit weniger Angriffsmöglichkeiten in der Lunge haben und daher weniger Virulenz für Personen aufweisen, die an große Höhen angepasst sind.

Der GHST liegt auf 1270 m Meereshöhe, die Patienten verbringen im Schnitt ca. 4 h dort

Der GHST liegt auf 1270 m Meereshöhe und die Patienten verbringen im Schnitt ca. 4 h dort. Es ist anzunehmen, dass in dieser Zeit physiologische Anpassungsprozesse, insbesondere bei wiederholten Einfahrten, stattfinden bei Menschen, die nicht an diese Meereshöhe adaptiert sind. Dies konnten Schobersberger und Mitarbeiter (2004) in ihrer Austrian Moderate Altitude Study zum Teil auch auf das Immunsystem zeigen.

## Conclusio

Der Übertragungs- und Infektionsmechanismus von Viren allgemein und von SARS-CoV‑2 ist komplex und noch nicht in allen Aspekten ausreichend erforscht. Es gibt jedoch bereits viele Befunde und Hinweise, wie oben dargestellt, die zeigen, dass eine Ansteckung im GHST mit Viren auf Grund der bestehenden klimatischen und physikalischen Bedingungen (hohe Luftfeuchtigkeit, Wärme, Radon) deutlich reduziert ist. In jeglicher Lebenssituation besteht eine Restgefahr, und es darf nicht vergessen werden, dass die Wirkfaktoren des GHST bei wiederholten therapeutischen Einfahrten, positive gesundheitliche Effekte haben.

Im Jahr 2018 führte Offenbächer und Kollegen einen Onlinesurvey mit Patienten mit entzündlich-rheumatischen (*N* = 503; [31]) und mit degenerativen Erkrankungen sowie mit einem chronischen Schmerzsyndrom (*N* = 368; [32]) durch, die regelmäßige Kuren durchführen. Die Befragten beider Patientengruppen stimmten zu bzw. stimmten überwiegend zu, dass durch regelmäßige Heilstollenkuren sich u. a. ihre Schmerzen reduzieren (in 89,5 % bzw. 84,8 %) und sich ihr Gesundheitszustand verbessert (in 92,1 % und 86,6 %).

Die positive Wirkung des GHST bestätigte sich auch in einer Untersuchung mit qualitativen Interviews (unveröffentlichte Daten). Acht Patienten mit entzündlich-rheumatischen Erkrankungen und sechs Patienten mit Fibromyalgie gaben an, dass sie durch regelmäßige therapeutische Besuche im GHST in der Lage waren, Medikamente (Schmerzmittel, Kortison, Biologika) zu reduzieren oder sogar abzusetzen. Die 14 Patienten berichteten ebenso über ausgeprägte Verbesserungen in einer Reihe von Symptomen (Schmerz, Steifigkeit, Depression und schlechte Schlafqualität) und funktionellen Einschränkungen. Sie zeigten sich überzeugt, nun über ein effektiveres Immunsystem zu verfügen (sie berichteten über weniger Infektepisoden), was auch auf die komplexen Effekte der milden Radonhyperthermie im GHST auf verschiedene physiologische Prozesse hindeutet.

Unserem Wissen nach gab es in der 68-jährigen Geschichte des GHST mit therapeutischen Einfahrten nie ein größeres Problem mit einer erhöhten Ansteckungsgefährdung, hohen Infektionsraten bzw. einer Epidemie, auch nicht in Zeiten, die durch eine Influenzawelle gekennzeichnet waren. Insgesamt überwiegt aus unserer Sicht der Nutzen der Heilstollentherapie bei weitem das Risiko einer möglichen Ansteckung durch Viren im GHST. Um dieses Risiko noch einmal zu reduzieren, wurden evidenzbasierte Präventionsmaßnahmen im GHST und im Kurhaus eingeführt, um eine Gefährdung der Patienten weiter zu minimieren. Zu diesen gehören u. a.: Informationen und Aufforderung zum Abstandhalten in jeder Phase im Kurhaus und Therapiebereich, Sprechverbot bei der Ein- und Ausfahrt, zusätzliche physikalische Barrieren im Zug und eine Temperaturmessung vor der Einfahrt.
